# Integrated Analysis of SNPs and Structural Variations via High-depth Whole-genome Sequencing Reveals the Genetic Architecture and Optimizes Genomic Prediction in Chickens

**DOI:** 10.1016/j.psj.2026.107561

**Published:** 2026-08-03

**Authors:** Haoqiang Ye, Siyu Zhang, Lin Qi, Xiaoqi Liu, Semiu Folaniyi Bello, Changbin Zhao, Wen Luo, Qinghua Nie

**Affiliations:** a Department of Animal Genetics, Breeding and Reproduction, College of Animal Science, South China Agricultural University, Guangzhou 510642, China; b Guangdong Provincial Key Lab of Agro-Animal Genomics and Molecular Breeding and Key Lab of Chicken Genetics, Breeding and Reproduction, Ministry of Agriculture and Rural Affair, South China Agricultural University, Guangzhou 510642, China; c Fujian Key Laboratory of Animal Genetics and Breeding, Institute of Animal Husbandry and Veterinary Medicine, Fujian Academy of Agricultural Sciences, Fuzhou, Fujian, 350013, China; d Agriculture Research Group, Organization of African Academic Doctors (OAAD), Off Kamiti Road, P. O. Box 25305-00100, Nairobi, Kenya

**Keywords:** Structural variation, Genome‐wide association analysis, Deep learning, Genomic prediction, Chicken

## Abstract

The characterization of genetic architecture and the optimization of genomic prediction are pivotal for the genetic improvement of complex traits in poultry. In this study, we investigated the genetic basis of 15 growth and carcass traits in an F2 chicken population (n = 877) using high-depth whole-genome sequencing with an average coverage of 31.2 × . By implementing an ensemble strategy involving four independent callers, we identified 35,924 high-confidence structural variations (SVs), with deletions being the most prevalent type. Combining SNPs and SVs enhanced genomic heritability for 14 out of 15 traits compared to SNPs alone. SNP-based GWAS corroborated well-known genes, including the prominent QTL cluster on chromosome 1, the *NCAPG-LCORL* locus on chromosome 4, and *IGF2BP1* on chromosome 27. Notably, SV-based analysis unveiled additional candidate genes, such as *ZNF385D, MYH10*, and *MOB1B*. To gain functional insights, eQTL-GWAS colocalization analysis integrating SNP-based GWAS signals with tissue-specific eQTL data identified significant colocalization signals for ITM2B in brain tissue, potentially implicating excitatory synaptic transmission, and TRIM13 in blood, potentially implicating inflammatory and immune regulation. To optimize genomic breeding value estimation through the effective utilization of multi-type markers, we developed GPDLBP, a hybrid deep learning framework that integrates locally connected networks to capture SV effects with the GBLUP model for SNP effects. Compared with the traditional SNP-only model, GPDLBP improved prediction accuracy for most traits, with gains exceeding 2% for BW21 (body weight at 21 days of age), BW49 (body weight at 49 days of age), EW (eviscerated weight), LMW (leg muscle weight), and AFW (abdominal fat weight); for example, prediction accuracy increased from 0.512 to 0.532 for BW49 and from 0.471 to 0.491 for EW. These findings show that SVs complement SNPs in both genetic dissection and genomic prediction of economically important traits in chickens. The integration of multiple variant types provides a practical strategy for accelerating precision breeding in high-depth sequencing-based poultry programs.

## Introduction

The use of DNA marker information has facilitated the development of genome research (Goodwin et al., 2016; Guan et al., 2025; Mardis and Wilson, 2025). As the most prevalent form of DNA markers, genome-wide single nucleotide polymorphisms (SNPs) allow researchers to estimate the genetic effects underlying complex traits by leveraging the linkage disequilibrium (LD) between SNPs and quantitative trait loci (QTLs) (Ren et al., 2022; Zhu et al., 2023). Based on the potential advantages of SNPs, the SNP array chip technology was developed and researchers subsequently applied them to breeding programs of agriculture. For instance, genomic prediction using SNP arrays to estimate genomic breeding values (GEBVs) has been widely implemented for the genetic improvement of livestock (Teissier et al., 2020; Pedrosa et al., 2024; Li et al., 2025), poultry (Wolc et al., 2015; Ye et al., 2023), and plants (Gamal El‐Dien et al., 2024), accelerating genetic progress. In addition, to better understand the underlying genetic architecture, some researchers employed genome-wide association study (GWAS) with SNPs to detect the QTLs and genes affecting, offering critical insights into the genetic basis of complex traits (Yang et al., 2021a; Zhao et al., 2021; Lu et al., 2021). In the realm of human genetics, these genomic strategies are widely applied to predict disease risk scores and dissect highly polygenic complex traits (Lewis and Vassos, 2020; Zheng et al., 2024). The comprehensive application of these genomic methodologies is expected to accelerate the trajectory of genetic research and its practical applications.

Driven by rapid advancement of whole-genome sequencing (WGS) technology and the decreasing cost of genome sequencing, using WGS alternative to SNP array has been widely adopted in genetic research (Levy and Myers, 2016; Lu et al., 2025). Compared to SNP array data, WGS data provides a higher density of SNPs that are more evenly distributed across the genome (Davey et al., 2011). Moreover, WGS data includes a vast number of genomic variants, enabling the transition from QTL detection to quantitative trait nucleotide (QTN) detection. This transition enhances the ability to directly identify causal variants, potentially improving the overall accuracy of genomic prediction (Zhu et al., 2023) and facilitating the detection power of rare causal variants (Cirulli and Goldstein, 2010). Additionally, high-depth WGS data can call high-quality structural variants (SV), which are large DNA segments, encompassing deletions, insertions, duplications, inversions, and translocations relative to the reference genome. SVs may theoretically exert a more profound impact on gene regulation and function compared to SNPs, as they can affect gene dosage or lead to alterations in gene structure (Cameron et al., 2019; Yang et al., 2024b; Ma et al., 2024). This allows SVs to capture extensive genomic changes, in contrast to the single base variations reflected by SNPs (Yang et al., 2024a). Furthermore, SVs have been implicated in contributing to the missing heritability of complex traits that cannot be fully explained by SNPs alone (Zhang et al., 2024), as not all SVs exhibit strong linkage disequilibrium (LD) with SNPs. Researchers have conducted SV-based association analyses on economic traits of hybrid pigs, revealing that SVs play a crucial role in determining body size (Zong et al., 2023). Similarly, in cattle, researchers identified SVs that have significant effects on cow production traits (Chen et al., 2021). However, some studies showed that incorporating SVs into the Genomic Best Linear Unbiased Prediction (GBLUP) model had a limited impact on improving genomic prediction accuracy (Chen et al., 2021; Zhuang et al., 2023). Therefore, it is necessary to explore how to effectively utilize SVs in genome prediction.

Machine learning (ML) is a powerful alternative approach for genomic prediction as it can construct complex non-linear models and effectively capture intricate non-linear effects (Costa et al., 2022; Chafai et al., 2023). As a result, ML has been recognized as a valuable tool for analyzing large-scale genomic datasets in genomic prediction, with many studies reporting the successful application of ML networks for breeding values prediction (Abdollahi-Arpanahi et al., 2020; Wang et al., 2022; Pedrosa et al., 2024). Deep learning (DL), a more advanced subset of ML, is inspired by the structure and function of the human brain and is based on a deep multi-layered neural network architecture where a large number of nodes and layers are constructed during network training (Eraslan et al., 2019). For large-scale genomic data, only using fully connected neural networks (FCNs) exhibit high computational complexity and are prone to overfitting. To address this issue, researchers have utilized convolutional neural networks (CNNs) to extract local genomic information more efficiently (Zingaretti et al., 2020). CNNs enhance computational efficiency by leveraging parameter sharing, where the same convolutional kernel operates on different local regions across the genome (Pook et al., 2020). However, genetic variants in different genomic regions contribute unequally to the genetic variance of traits. Variants with similar sequence features may exert distinct functional effects depending on their genomic context. To improve biological interpretability, researchers have used locally connected neural networks (LCNNs) (Lee et al., 2023), which is similar to CNN but removes the parameter sharing mechanism, allowing the weights of each local region to be learned independently. This approach is better suited to capture the functional heterogeneity of different genomic regions.

Poultry products are a major globally traded commodity and a vital source of animal protein, addressing the escalating consumer demand. Although GWAS and GS studies in chickens have increased recently (Yang et al., 2021b; Liu et al., 2022; Zhong et al., 2024), most have focused solely on SNPs, while research on SVs remains largely understudied. This gap is primarily due to the prevalence of SNP array data imputed to the sequence level or low-depth sequencing in current livestock genomics studies, both of which lack the resolution required for high-quality SV calling. Our current study systematically identified SNPs and SVs from the high-depth WGS data in chicken, providing a theoretical basis for analyzing the genomic features of these two marker types. The study conducted a comprehensive evaluation of LD, narrow-sense heritability, genome-wide association analysis and genomic prediction performance across SNPs, SVs, and their combined application. Furthermore, we proposed a novel hybrid algorithm for genomic prediction that integrated two distinct marker variant types (SVs and SNPs). The framework synergistically combined DL networks with the GBLUP model, where the DL component, using LCNNs, was designed to specifically capture genetic effects of SVs, while the GBLUP module accounted for genetic effects derived from SNPs. Detailed implementation details of the algorithm framework are available in the Methods section. The objective of this study was to provide new insights into the genetic architecture of economic traits in chickens by integrating SVs and SNPs, as well as to develop a novel strategy for genomic prediction utilizing both marker variant types.

## Materials and methods

### Population and phenotypes

The experimental population utilized in this study consisted of an F2 chicken population generated from intercrosses between the Xinghua chicken (an indigenous Chinese breed) and a Recessive White Plymouth Lock chicken line. The chickens are provided and managed by the Wens Nanfang Poultry Breeding Co. Ltd. (Xinxing, PR China). All experimental individuals were raised on the same farm under uniform feeding and management protocols. Phenotypic data for 15 traits were systematically collected and utilized for the analyses presented. Phenotypic measurements covered 3 growth traits (body weight at hatch (BW1), body weight at 21 days of age (BW21) and body weight at 49 days of age (BW49)) and 12 carcass traits (breast muscle weight (BMW), leg muscle weight (LMW), half eviscerated weight (HEW), eviscerated weight (EW), wing weight (WW), abdominal fat weight (AFW), pre-slaughter live weight (PSLW; body weight measured immediately before slaughter), slaughter weight (SW; carcass weight measured after slaughter), head and neck weight (HNW), shank and claw weight (SCW), heart-liver-gizzard-glandular-stomach weight (HLGGSWs), and small intestine length (SIL)). All individuals were slaughtered at 90 days of age. The detailed data of these traits are shown in Additional file Table S1.

### Whole genome sequencing, variant detection and quality control

High-depth whole-genome resequencing was performed on the experimental F2 population (n = 877) derived from a cross between Xinghua and Recessive White chickens, consisting of 162 full-sibs and 715 half-sibs. For each individual, 2 mL of blood was collected from the subcutaneous vein for genomic DNA extraction and subsequent library preparation (Adey et al., 2010). Sequencing was conducted on the DEBSEQ-T7 platform (BGI, Shenzhen, China), achieving a mean coverage depth of 31.2 × across the entire population.

The sequencing reads generated from the libraries were first processed for quality control using fastp (v0.20.0) (Chen et al., 2018) with default parameters. The resulting clean reads were then aligned to the chicken reference genome (bGalGal1.mat.broiler.GRCg7b) using BWA (v.0.7.17) (Li and Durbin, 2009). SAMtools (v.1.17) (Li et al., 2009) was employed to convert file formats, sort the alignments, and create indices. Duplicated reads were marked and removed using PicardTools (DePristo et al., 2011). This pipeline yielded final aligned BAM files.

SNP calling was performed using the GATK (DePristo et al., 2011) HaplotypeCaller module, generating individual GVCF files from the aligned BAM files. Subsequently, these GVCFs were imported into a GenomicsDB using the GATK GenomicsDBImport module, followed by joint genotyping with GATK GenotypeGVCFs to produce the final VCF file. Variant filtering was then implemented with GATK VariantFiltration for the selected SNPs, applying the following parameters: QD < 2.0 | MQ < 40.0 | FS > 60.0 | SOR > 3.0 | MQRankSum < -12.5 | ReadPosRankSum < -8.0. Non-biallelic SNPs were excluded. Genotype imputation was performed using Beagle5.4 software (default parameters). No external reference panel was used; therefore, imputation was performed solely to recover sporadically missing genotypes, rather than to infer previously untyped variants. Following imputation, SNPs with a minor allele frequency (MAF) < 0.05 were excluded using PLINK (v1.9) (Chang et al., 2015) to ensure adequate minor-allele representation. This filtering retained 13,465,449 variants for subsequent analyses. Specifically for genomic prediction, the dataset was further refined through linkage disequilibrium (LD) pruning (–indep-pairwise 50 5 0.9), yielding a subset of 6,809,336 variants to ensure efficient processing. LD pruning was applied to reduce marker redundancy and computational burden in genomic prediction.

Structural variants (SVs), including deletions (DELs), inversions (INVs), duplications (DUPs), and translocations (TRAs), were independently detected in each sample using four tools: DELLY (v0.0.1) (Rausch et al., 2012), Smoove (v0.2.7; https://github.com/brentp/smoove), Manta (v1.6.0) (Chen et al., 2016), and CNVnator (v0.3.3) (Abyzov et al., 2011). All tools were run using their default parameters. On average, DELLY, Smoove, Manta, and CNVnator detected 28,160, 4,708, 11,562, and 2,377 SVs per sample, respectively (Table S2). Within each sample, the caller-specific SV sets were merged using SURVIVOR (v1.0.7) (Jeffares et al., 2017) with the parameters 1000 2 1 1 0 50. Accordingly, an SV was required to be supported by at least two callers, with agreement in SV type and strand orientation. A maximum breakpoint distance of 1,000 bp was applied uniformly across SV types and sizes, and the minimum SV length was set to 50 bp.

After within-sample merging, SVs were genotyped separately for each individual. The resulting individual-level datasets were subsequently merged across samples using Jasmine software (https://github.com/jasmine/jasmine). Site-level QUAL was evaluated after population-level merging rather than by directly comparing caller-specific quality scores. SV sites were retained if they were detected in at least one individual and met the following criteria: SV length > 50 bp and site-level QUAL> 200. This filtering yielded a final set of 35,924 SVs for downstream analyses.

### Genome-wide enrichment analysis of genes and structural variants

To evaluate whether annotated genes and structural variants (SVs) were preferentially distributed in specific genomic regions, we performed within-chromosome permutation analyses using the regioneR package. Chromosome-end regions were operationally defined as the terminal 5% of chromosome length at each end of every chromosome; thus, the two chromosome-end regions together accounted for 10% of each chromosome.

To define gene-rich and gene-poor regions, the genome was divided into non-overlapping 1-Mb windows. Each annotated gene was represented by its midpoint and assigned to a single window according to the midpoint position, thereby preventing genes spanning adjacent windows from being counted more than once. Gene density was calculated as the number of gene midpoints per megabase of callable sequence within each window. Among the windows, those with gene density at or above the genome-wide 75th percentile were classified as gene-rich, whereas those with gene density at or below the 25th percentile were classified as gene-poor. The remaining windows were classified as regions of intermediate gene density.

The test statistic was the number of features overlapping the target regions. A total of 10,000 permutations were performed for each comparison. The expected overlap count was calculated as the mean of the permuted overlap counts, and fold enrichment was calculated as the ratio of the observed overlap count to the expected overlap count. Empirical P values were obtained from the permutation distributions. P values from all enrichment tests were adjusted for multiple comparisons using the Benjamini–Hochberg false discovery rate (FDR) procedure.

### Population structure and Linkage disequilibrium analysis

To account for population structure and relatedness, SNP-based and SV-based genomic relationship matrices (GRMs) were constructed separately using GCTA (Yang et al., 2015) following the methodology proposed by Meuwissen et al (Meuwissen et al., 2001). For SV-GRM construction, all retained SVs were treated as biallelic diploid markers and encoded as 0, 1, and 2 according to the number of alternative alleles. Principal Component Analysis (PCA) was performed separately on the SNPs and SVs using PLINK (v1.9) software.

Investigation of LD involving SVs was conducted referring to previous research with little modifications (Yan et al., 2024). Pairwise genotype LD (coefficient of determination, $R^{2}$ value) was calculated for all complete cases of both SNP-SV and SNP-SNP pairs using PLINK (v1.9) with default parameter settings. For SNP-SV pairs, we defined genomic regions by extending 5 kb upstream and 5 kb downstream from each SV. SNPs within these regions were then combined with the respective SV into a single input file for PLINK to calculate $R^{2}$ values. The output file contained the $R^{2}$ value for each SNP-SV pair. For SNP-SNP pairs, our analysis focused exclusively on SNPs located within the same 5 kb upstream and 5 kb downstream windows flanking each SV. Specifically, for each SV, SNPs within these defined flanking regions were extracted into a file, and pairwise $R^{2}$ values were calculated among them. The output file provided $R^{2}$ values for every SNP-SNP pair within these windows. To classify SVs based on their LD patterns, the median $R^{2}$ value across all SNP-SNP pairs was first determined. Subsequently, for each SV, we calculated the frequency at which its associated SV-SNP $R^{2}$ values exceeded this overall median SNP-SNP $R^{2}$ value. SVs with a frequency less than 0.5 were categorized as relatively low-LD SVs, while those with a frequency greater than 0.5 were classified as relatively high-LD SVs.

### Estimation of genetic parameter

The *reml* module of GCTA software (Yang et al., 2015) was used for variance component estimation and heritability calculations of 15 traits. Specifically, based on the phenotype and genome information, variance components were estimated using the single-trait genomic best linear unbiased prediction model.

The statistical model is:

y=Xb+Zg+e

Where y is a vector of phenotype value; b is the vector of fixed effects, including sex (with two levels) and batch (with eight levels); g is the vector of the additive genetic values explained by the genetic variants (SNPs or SVs), following a assumed distribution of $N(0,G\sigma_{g}^{2})$, where $\sigma_{g}^{2}$ is the additive genetic variance and G is the individual genomic relationship matrix derived from SNPs or SVs; e is the vector of random residual effects with assumed distribution of $N(0,I\sigma_{e}^{2})$, where $\sigma_{e}^{2}$ is the residual variance and I is the identity matrix; X and Z are design matrices for b and g, respectively. The heritability was calculated by $h^{2}=\sigma_{g}^{2}/\left(\sigma_{g}^{2}+\sigma_{e}^{2}\right)$.

We also estimate the heritability using two-component genomic best linear unbiased prediction model via the formula:

$$y=Xb+Zg_{snp}+Zg_{sv}+e$$

Where y, X, b, Z, e are the same as above; $g_{snp}$ is the vector of the additive genetic values captured by SNPs, following a assumed distribution of $N(0,G\sigma_{g_{SNP}}^{2})$, where $\sigma_{g_{SNP}}^{2}$ is the additive genetic variance explained by SNPs; $g_{sv}$ is the vector of the additive genetic values captured by SVs, following a assumed distribution of $N(0,G\sigma_{g_{SV}}^{2})$, where $\sigma_{g_{SV}}^{2}$ is the additive genetic variance explained by SVs. In this context, the heritability was calculated by $h^{2}=\left(\sigma_{g_{SNP}}^{2}+\sigma_{g_{SV}}^{2}\right)/\left(\sigma_{g_{SNP}}^{2}+\sigma_{g_{SV}}^{2}+\sigma_{e}^{2}\right)$.

We further estimated the genetic correlation between pairs of 15 traits using the *reml-bivar* module of GCTA. The genetic correlation was estimated using two-trait genomic best linear unbiased prediction model, which is formulated as follows:

$$\left[\begin{array}{c} y_{1}\\ y_{2} \end{array}\right]=\;\left[\begin{array}{cc} X_{1} & 0\\ 0 & X_{2} \end{array}\right]\left[\begin{array}{c} b_{1}\\ b_{2} \end{array}\right]+\left[\begin{array}{cc} Z_{1} & 0\\ 0 & Z_{2} \end{array}\right]\left[\begin{array}{c} g_{1}\\ g_{2} \end{array}\right]+\left[\begin{array}{c} e_{1}\\ e_{2} \end{array}\right]$$

Where $\left[\begin{array}{c} y_{1}\\ y_{2} \end{array}\right]$ is the vector of phenotype values for the pairs of traits; $\left[\begin{array}{c} b_{1}\\ b_{2} \end{array}\right]$ is the vector of fixed effects, including sex and batch; $\left[\begin{array}{c} g_{1}\\ g_{2} \end{array}\right]∼N\left(0,G[\begin{array}{cc} \sigma_{g_{1}}^{2} & \sigma_{g_{12}}\\ \sigma_{g_{21}} & \sigma_{g_{2}}^{2} \end{array}]\right)$ is the vector of additive genetic effects explained by the genetic variants (SNPs or SVs), where $\left[\begin{array}{cc} \sigma_{g_{1}}^{2} & \sigma_{g_{12}}\\ \sigma_{g_{21}} & \sigma_{g_{2}}^{2} \end{array}\right]$ is the additive genetic variance-covariance matrix and G is the individual genomic relationship matrix derived from SNPs or SVs; $\left[\begin{array}{c} e_{1}\\ e_{2} \end{array}\right]∼N\left(0,I[\begin{array}{cc} \sigma_{e_{1}}^{2} & \sigma_{e_{12}}\\ \sigma_{e_{21}} & \sigma_{e_{2}}^{2} \end{array}]\right)$ is the vector of random residual effects, where $\left[\begin{array}{cc} \sigma_{e_{1}}^{2} & \sigma_{e_{12}}\\ \sigma_{e_{21}} & \sigma_{e_{2}}^{2} \end{array}\right]$ is the residual variance-covariance matrix; $\left[\begin{array}{cc} X_{1} & 0\\ 0 & X_{2} \end{array}\right]$ and $\left[\begin{array}{cc} Z_{1} & 0\\ 0 & Z_{2} \end{array}\right]$ are the corresponding design matrices for the $\left[\begin{array}{c} b_{1}\\ b_{2} \end{array}\right]$ and $\left[\begin{array}{c} g_{1}\\ g_{2} \end{array}\right]$, respectively. Variance components and their standard errors (SEs) were estimated using the average information restricted maximum likelihood (AI-REML) algorithm. Based on estimated genetic correlations, we conducted a hierarchical cluster analysis for these traits.

### Single-trait genome-wide association analysis

In this study, we performed a single-trait GWAS by implementing a mixed linear model in the GCTA software. The association test for each variant (both SNPs and SVs) was based on the following model:

y=Wa+xc+Xb+u+e

Where y represents the phenotype vector; W is the indicator matrix of the fixed effects (i.e., sex and batch); a is the vector of corresponding fixed effects; c indicates the covariates, including the top five PCs based on SNPs; x represents the regression coefficient of covariates; X is the vector of variant genotypes at the locus tested; b is the corresponding effect of the marker; $u∼N(0,KV_{g})$ is the vector of random polygenic effects, in which K is the kinship matrix derived from SNPs markers and $V_{g}$ is the polygenic additive variance; $e∼N(0,IV_{e})$ is the vector of random residual, in which I is the identity matrix and $V_{e}$ is the residual variance.

### Multi-traits genome-wide association analysis

To leverage the shared genetic architecture across multiple phenotypes, we conducted a multi-trait analysis using the C-GWAS framework (Xiong et al., 2022). This method performs a joint analysis by integrating summary-level statistics derived from single-trait GWASs, allowing for the effective examination of potentially correlated traits. We compared this method with the conventional approach, which involves conducting separate GWASs for each trait and assessing significance using the minimum p-values of multiple GWASs (MinGWAS) with a more stringent significance threshold. Following the positive findings by CGWAS, we applied statistical indicators to quantify the multi-trait effect (MTE) (Xiong et al., 2022), which used the skewness of the independent phenotypic effects of each marker to represent its multi-trait effect. In this context, a lower skewness value indicates that a SNP is associated with a greater number of independent phenotypes, which in turn corresponds to a larger multi-trait effect. The opposition of the centralized skewness of each SNP is considered as a quantification of its multi-trait effect, $MTE=2\sqrt{2}-Skew\left(pt\circ pt\right)$, where pt is phenotypic effects for each significant marker. MTE < 0 suggests insufficient evidence to support the presence of multi trait effect (Xiong et al., 2022).

### Gene-based association analysis

To examine the pleiotropy of genes in related traits at the gene level, we performed gene-based association analysis based on C-GWAS summary statistics using MAGMA (De Leeuw et al., 2016). First, we integrated the genomic annotation of the chicken reference genome (bGalGal1.mat.broiler.GRCg7b) with the marker genotypes information and annotated SNPs and SVs according to each gene’s genomic position. Subsequently, gene-level association analysis was performed using annotation information to compute the associations at the gene level.

### Colocalization analysis

To evaluate whether a shared underlying variant drives both GWAS signals and gene expression within specific genomic regions, we performed Bayesian colocalization analysis using the R package 'coloc' (Giambartolomei et al., 2014). The reference genomic coordinates were converted from the GRCg7b assembly to GRCg6a using the liftOver tool (http://genome.ucsc.edu/cgi‐bin/hgLiftOver) to ensure compatibility across datasets. Further, we integrated the C-GWAS-identified loci with eQTL datasets from 28 tissues available in the Chicken GTEx resource (Guan et al., 2025). The standard coloc framework assumes at most one causal variant per trait within each tested region. The analysis encompassed regions spanning 1 Mb upstream and downstream of the regional lead SNPs identified in C-GWAS. Under the Bayesian framework with default prior probabilities, the posterior probability of hypothesis 4 (PP4) greater than 0.85 was considered strong evidence of colocalization.

### Functional annotation

To investigate the potential biological functions of the significant variants, we conducted functional annotation on the positive findings and identified candidate genes using gene information annotated by Ensemble dataset. The candidate genes were subjected to pathway enrichment analysis against the Kyoto Encyclopedia of Genes and Genomes (KEGG) database to identify the biological pathways. The enrichment analysis was performed using the R package ‘clusterProfiler’ (Wu et al., 2021).

### Genomic prediction

Genomic estimated breeding values (GEBVs) were predicted using the Genomic Best Linear Unbiased Prediction (GBLUP) model implemented in the HIBLUP software (Yin et al., 2023). For genomic prediction based on either SNPs or SVs separately, a single-component GBLUP model was applied, as described below:

y=Xb+Zg+e

where the notation and variable definitions are consistent with Equation (1).

For the joint application of SNPs and SVs, a two-component GBLUP model was employed for genomic prediction, modeled as follows:

$$y=Xb+Zg_{snp}+Zg_{sv}+e$$

where the notation and variable definitions follow those in Equation (2).

The present study proposes GPDLBP, a novel genomic prediction method that integrates genomic-prior deep learning locally connected networks and the best linear unbiased prediction (GBLUP) framework. GPDLBP integrates two distinct marker variant types (SVs and SNPs) by synergistically combining DL networks with the GBLUP model, where the DL component is designed to specifically capture genetic effects of SVs, while the GBLUP module accounts for genetic effects derived from SNPs. The procedure of GPDLBP is performed as follows:

$${\hat{y}}_{cor}=u+\;g_{deep}+\;w_{a}{\hat{g}}_{a}\;+e$$

where ${\hat{y}}_{cor}$ is the corrected phenotype; u is the mean term; ${\hat{g}}_{a}$ is the additive genomic estimated breeding values derived from SNPs using a single-component GBLUP model; $g_{deep}$ denotes the genomic breeding values estimated from structural variants (SVs) using a deep learning approach;e is the residual vector; $w_{a}$ is the weights assigned to$\;{\hat{g}}_{a}$. In the following, we provide a detailed description of the deep learning process used to estimate $g_{deep}$.

Step 1: Within each outer training fold, a two-component GBLUP model was first fitted using SNP- and SV-derived genomic relationship matrices:
  $$y=Xb+Z_{SV}g_{SV}+\;Z_{SNP}g_{SNP}+e$$

where the symbols are described as above. Only phenotypic observations from the current outer training population were included in model fitting.

Step 2: Estimate the variance contribution of each SV based on ${\hat{g}}_{sv}\;$derived from SVs. First, the genetic effect of each SV is estimated using a specified formula:
  $$\hat{a}=Z^{\prime }G^{-1}{\hat{g}}_{sv}$$

and then its genetic variance contribution is calculated based on its genetic effect and allele frequency, as follows:

$$d_{i}=\;{\hat{a}}_{i}^{2}*2p_{i}\left(1-p_{i}\right)$$

where $d_{i}$ represents the genetic variance contribution of $SV_{i},\;$and $p_{i}$ denotes its minor allele frequency.

Step 3: Extract SVs with variance contributions greater than the average, referred to as QTL_SVs (prior QTL-associated SVs), and model their genetic effects using the deep neural network. Here, QTL_SVs denotes a training-specific, statistically prioritized subset of trait-associated candidate SVs based on estimated variance contributions and does not imply that these variants are confirmed causal QTLs.

In the input layer, we construct a locally connected neural network layer based on prior genomic information within each training fold. First, grouping the QTL_SVs based on their variance contributions, such that those with similar contributions are assigned to the same neuron group. The number of groups, denoted as k (i.e., neurons), is determined by dividing the total number of QTL_SVs by the parameter n (kernel size), meaning that every n QTL_SVs with the most similar variance contributions are clustered into a single neuron. In this study, the parameter n was set to 10. Let $t\;\in \;R^{p}$ and $o\;\in \;R^{k}$ be the input and output feature vectors, and the proposed genome prior LCL calculates the *i-*th value $o_{i}$ of the output o as follows:

$$o_{i}=\;\sum_{j=1}^{n}w_{i,j}t_{i,j}$$

where $w_{i,j}$ is the *j*-th kernel weight for the *i-*th output in trainable weight matrix $W\;\in \;R^{K*N}$. Therefore, the LCL operation can be written as:

$$o=LCL^{k,n}\left(t,W\right)=\;\left[\sum_{j=1}^{n}w_{1,j}t_{1,j},\sum_{j=1}^{n}w_{2,j}t_{2,j},\ldots ,\sum_{j=1}^{n}w_{k,j}t_{k,j}\right]$$

Let $x_{l}\;$be the QTL_SV genotype of the *l*-th individual, the GPDLBP first captures the QTL_SV effects of the *l*-th individual through $LCL^{k,n}\left(x_{l},\cdot \right)$, followed by normalization and non-linear transformation, and then the output of this layer ${\tilde{e}}_{l}\in \;R^{k}$ can be derived from:

$${\tilde{e}}_{l}=\;GeLU\left(LN\left(LCL^{k,n}\left(x_{l},W_{\tilde{e}}\right)\right)\right)$$

where LN(·) is the function of layer normalization (Ba et al., 2016), GeLU(·) is the function of non-linear transformation (Hendrycks and Gimpel, 2023), and $W_{\tilde{e}}$ is the trainable weight of $LCL^{k,n}\left(x_{l},\cdot \right)$. Then, the transformed output is used as the input for the next network layer, where a conventional LCL with a kernel size of 5 is applied to model and compute the final genetic effects $e_{l}$:

$$e_{l}=\;LCL^{5}\left({\tilde{e}}_{l},W_{e}\right)$$

where $W_{e}$ is the trainable weight of $LCL^{5}\left({\tilde{e}}_{l},\cdot \right)$. To ensure the reusability of input sequences and maintain the stability of residuals, GPDLBP adds $e_{l}$ to residual layer (He et al., 2015):

$${\ddot{e}}_{l}=F\left(x_{l},W_{F}\right)+e_{l}$$

where $F(x_{l},\cdot )$ is a linear layer projection of the input $x_{l}$, and $W_{F}$ is the weight parameter of the linear projection.

Finally, GPDLBP estimates genomic breeding values$\;{\hat{g}}_{deep}$ from $e_{l}$ through a fully connected layer (FCL):

$${\hat{g}}_{\text{deep}\;}=FCL\left({\ddot{e}}_{l},W_{g}\right)$$

where $W_{g}$ is the trainable weight of $FCL(e_{l},\cdot )$.

Finally, the GPDLBP predicted genomic breeding values are given by:

$${\hat{y}}_{pre}=\;u+\;g_{deep}+\;w_{a}{\hat{g}}_{a}\;=f_{\theta }\left(x_{i,QTL-SV}\right)+w_{a}{\hat{g}}_{a}=Fun\left(W_{\tilde{e}},W_{e},W_{F},W_{g}\;\right)+\;w_{a}{\hat{g}}_{a}$$

Where $x_{i,QTL-SV}$ denotes the genotype vector of individual iat the variance-prioritized SVs selected exclusively from the corresponding training fold; $f_{\theta }\left(\cdot \right)$ denotes the deep-learning function parameterized by θ, including the trainable parameters of the locally connected layers, linear projection layer, normalization layer, and fully connected output layer; Fun(·) is the DL function. Accordingly, the proposed GPDLBP framework iteratively optimized the set of weights$\tilde{W}=\left\{W_{\tilde{e}},W_{e},W_{g},W_{F},w_{a}\right\}$ to minimize the loss function during the training. The mean squared error (MSE) was used as the loss function, calculated as the L2-loss between observed and predicted phenotypes. The AdamW optimizer (Loshchilov and Hutter, 2019) was employed to update model parameters throughout the training process. To prevent overfitting, Elastic Net penalty was added to the loss function:

$$L=\;\frac{1}{N}\sum_{i=1}^{N}∥y_{train}^{i}-{\hat{y}}_{train}^{i}{∥}_{2}^{2}+\omega_{1}∥\tilde{W}{∥}_{1}+\omega_{2}{∥\tilde{W}∥}_{2}^{2}$$

where $\omega_{1}$ and $\omega_{2}$ are the regularization weights for the L1 and L2 penalties, respectively. Both are set to 0.0001. For comparison, we also performed genomic prediction using a two-component GBLUP model based on SNPs and the QTL_SVs derived from the GPDLBP framework.

To assess the prediction performance of the models, we implemented a random 10-fold cross-validation scheme, replicated five times. In each repetition, individuals were randomly divided into ten mutually exclusive folds, with nine folds used as the outer training population and the remaining fold used exclusively as the outer validation population. All phenotype-dependent procedures, including variance-component estimation, SV-effect back-solving, SV variance-contribution ranking, SV selection, SNP-based GEBV estimation, deep-learning model fitting, and hyperparameter selection, were performed independently within each outer training fold. The parameters were estimated from the outer training individuals only and were subsequently applied to the corresponding validation individuals. The validation phenotypes were not used for feature selection and model training. The performance of prediction models was defined as Pearson’s correlation coefficient between the GEBVs and the corresponding adjusted phenotypic values in the validation population and averaged over the cross-validation.

## Results

### The landscape-wide SV discovery in the genomes of chicken

**Fig. 1:**
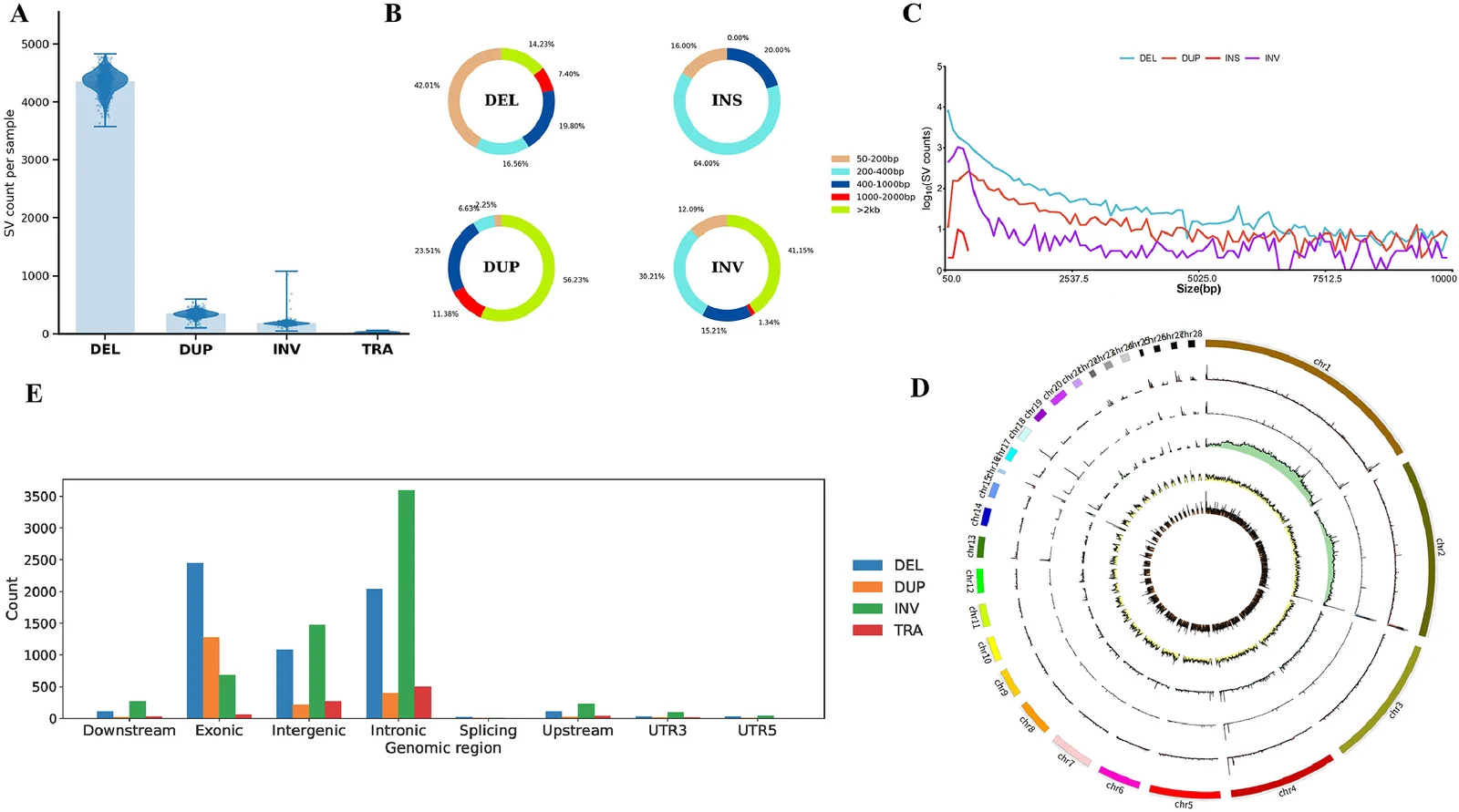
The landscape of structural variations in the chicken genome. **A:** Summary of SV counts per individual across four major types (DEL, DUP, INV, and TRA), with violin plots illustrating the distribution and density of variations. **B:** Doughnut charts represent the proportional distribution of five length categories for each SV type. **C:** Size distribution of SVs, with both x-axis (length) and y-axis (frequency) presented on a log10 scale. **D:** Genome-wide distribution of the discovered SVs in the chicken genome. The circle diagram shows the distribution of SVs in the chromosome, where concentric circles show the following from outside to inside: DELs, DUPs, INVs, SNPs, genes. E: Functional annotation of SVs across different genomic regions, categorized by SV type.

To improve the accuracy and sensitivity of SV detection, we applied four complementary callers-DELLY (v0.0.1), Smoove (v0.2.7), Manta (v1.6.0), and CNVnator (v0.3.3)-which implement distinct algorithmic strategies. After these quality-control and filtering steps, we identified 35,924 high-confidence SVs including 24,005 deletions (DELs), 4,615 duplications (DUPs), 25 insertions (INSs), 6,351 inversions (INVs), and 928 translocations (TRAs) (Fig. S1). Fig. 1A summarizes per-individual detection patterns, with an average of 4,348 DELs, 344 DUPs, 184 INVs, and 35 TRAs detected per individual. Due to inherent limitations of short-read-based SV discovery, INS detection was limited and TRA lengths could not be reliably inferred from breakpoint coordinates. Therefore, we further characterized the length distributions of DELs, DUPs, INSs, and INVs (Fig. 1B,C). Overall, SV counts decreased with increasing event size, and the length profiles differed markedly among SV classes. DELs were the most abundant and were enriched for short events (50-200 bp), while still exhibiting substantial representation in mid-length ranges. INSs were predominantly 200-400 bp and rarely exceeded 1 kb. Contrarily, DUPs were biased toward long events, with >2 kb accounting for the largest proportion and appreciable numbers remaining in mid-to-long size bins. INVs showed a comparatively broader distribution but were also enriched for >2 kb events; their abundance declined with size and displayed greater variability across bins.

We further examined the genome-wide distributions of variants and annotated genes. The density profiles in Fig. 1D suggested that annotated genes were more concentrated toward chromosome ends, whereas several SNP-dense segments coincided with regions of relatively low gene density. SVs exhibited heterogeneous genomic distributions, with local density peaks differing among SV classes and frequently occurring in gene-rich or chromosome-end regions.

Chromosome-constrained permutation analyses supported these descriptive patterns. Annotated genes were significantly enriched in chromosome-end regions (fold enrichment = 1.36, FDR < 0.001; Fig. S2). SVs overall were also enriched in chromosome-end regions (fold enrichment = 1.74, empirical $P<1.0\;\times 10^{-4}$). Significant enrichment was detected for deletions, duplications, and inversions, with duplications showing the strongest effect (fold enrichment = 3.36). Insertions showed no significant enrichment, although only 25 insertions were available for analysis. SVs overall were also modestly enriched in gene-rich regions (fold enrichment = 1.17, empirical FDR < 0.001). This enrichment was significant for deletions, duplications, and inversions, and was again strongest for duplications (fold enrichment = 1.38). Insertions were not significantly enriched in gene-rich regions. None of the SV classes showed evidence of enrichment in gene-poor regions in the one-sided enrichment tests.

Functional annotation revealed clear regional preferences for SV occurrence: most SVs localized in intronic and intergenic regions, with a smaller but detectable fraction in exonic regions, whereas splice-site–associated events were rare. Among SV classes, INVs were most concentrated in intronic regions and substantially outnumbered other types; DELs were abundant in both intronic and exonic regions; DUPs were relatively enriched in exonic regions; and TRAs were the least frequent overall, occurring mainly in intronic and intergenic regions (Fig. 1E).

### Population structure and linkage disequilibrium analyses using SVs and SNPs

To better elucidate the population structure among the studied chicken populations based on different maker types, we performed the principal components analysis (PCA) and genomic kinship analysis using both SNPs and SVs independently. In general, the SV-based PCA demonstrated tighter clustering patterns and reduced intra-cluster dispersion relative to the SNP-based analysis (Fig. S3 and B). A consistent pattern of pairwise genetic relatedness was observed in the kinship analysis, where the heatmap patterns derived from SNPs (Fig. S3C) and SVs (Fig. S3D) showed remarkable similarity. Both marker types consistently identified the block-like structures, which represent closely related subgroups. We assessed the LD between SVs and their flanking SNPs relative to the local SNP-SNP LD background. The results revealed that 93.6% of SVs exhibited lower LD with nearby SNPs compared to the LD derived solely from the nearby SNPs (Fig. S3E). Furthermore, SVs in the relatively high LD group possessed significantly higher minor allele frequencies (MAF) than those in the low LD group (Fig. S3F). The distributions of direct LD ($R^{2}$) between structural variants and nearby SNPs were present in Fig. S4.

### Heritability estimation using SNPs and SVs

**Fig. 2:**
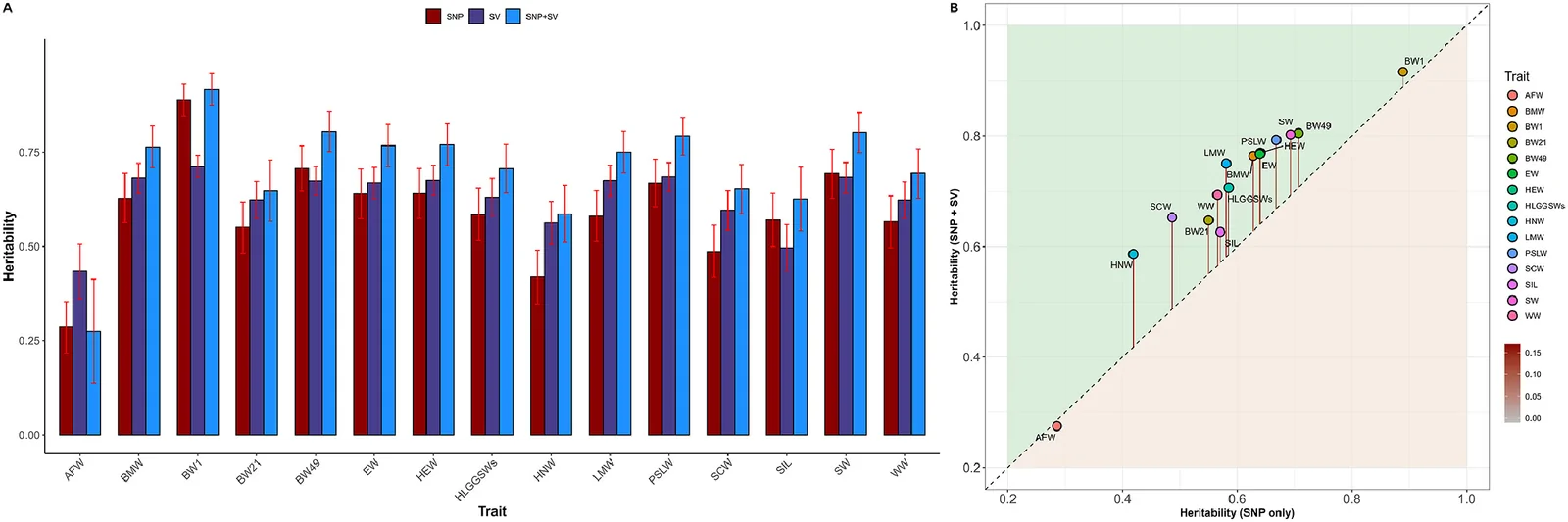
Genomic heritability estimation of 15 traits using SNP and SV markers. **A:** Heritability captured by different marker sets. The bar plot compares the genomic heritability estimated using SNPs only, SVs only, and the combined SNP+SV set for 15 quantitative traits. Error bars represent the standard error (SE) of the estimates. **B:** Comparative gain in heritability by incorporating SVs. The scatter plot illustrates the relationship between heritability estimated using SNPs only (x-axis) versus the combined SNP+SV model (y-axis). The dashed diagonal line represents y=x. The vertical lines and the color scale represent the magnitude of heritability improvement.

The genomic heritability of 15 traits using SNPs only, SVs only, and a combination of both (SNP+SV) was estimated. As shown in Fig. 2A, among the analyzed traits, AFW exhibited the lowest estimated heritability, while BW1 showed the highest. For most of the traits, heritability estimates ranged between 0.4 and 0.7. A comparison of using a single type of marker (SNPs or SVs) revealed distinct patterns in heritability estimation. SNPs yielded higher heritability estimates for BW1, BW49, SIL, and SW, whereas SVs produced higher estimates for the remaining traits. Notably, as shown in Fig. 2B, combining SNPs and SVs resulted in higher heritability estimates compared to using SNPs alone for 14 out of the 15 traits, where 14 traits are located above the diagonal in the green area. Specifically, LMW, HNW, and SCW showed the most substantial improvements, with increases values of 0.169, 0.168, and 0.166, respectively. Furthermore, even for traits like BW1 and BW49, where the SNP heritability estimates were already high (>0.7), the inclusion of SVs (SNP+SV) further enhanced the estimates (from 0.889 to 0.916 for BW1, and from 0.707 to 0.805 for BW49).

### Genetic correlation estimation and cluster analysis

**Fig. 3:**
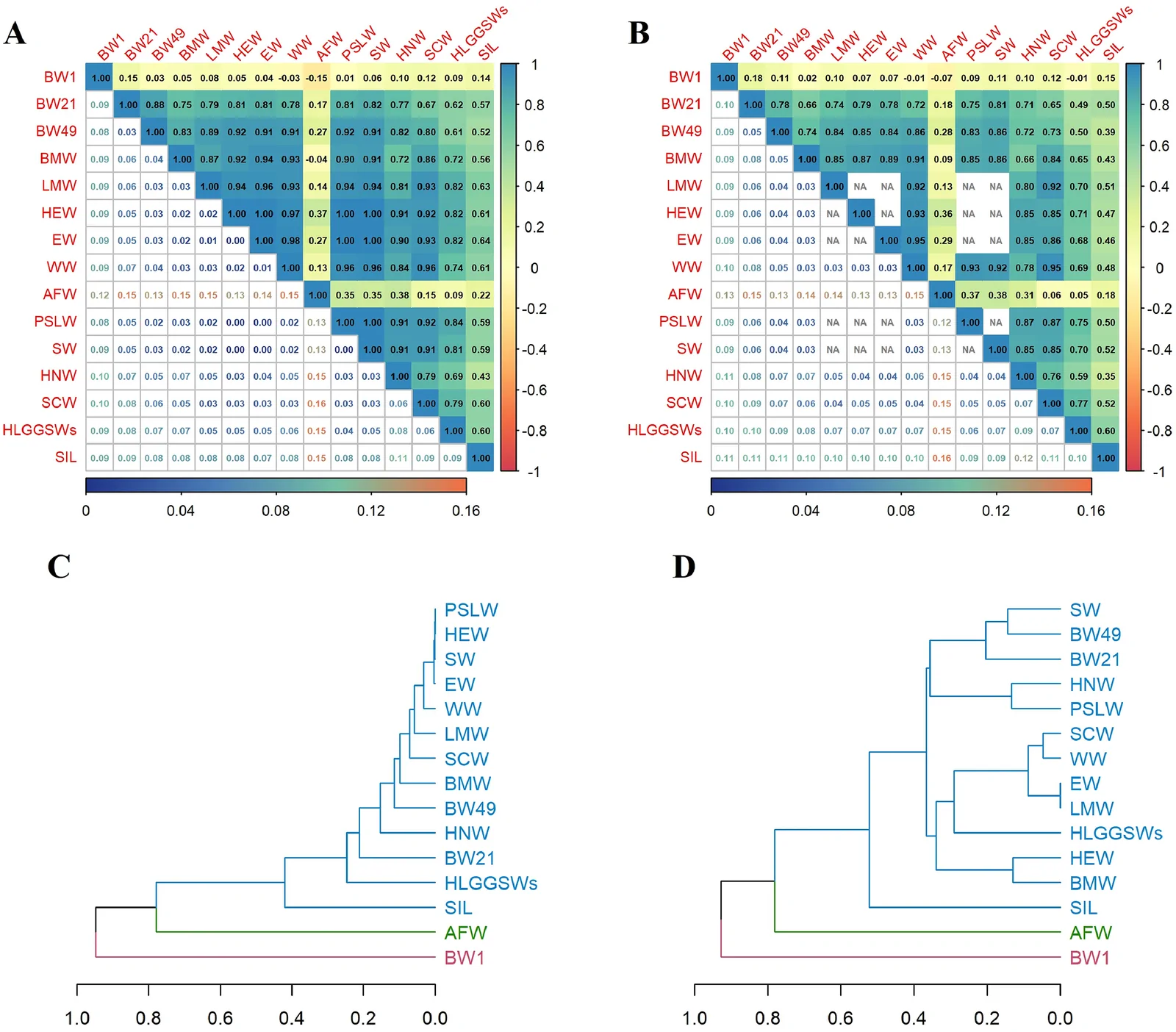
Pairwise genetic correlations and hierarchical clustering of 15 traits based on SNPs and SVs. **A & B:** Heatmaps illustrating pairwise genetic correlations estimated using SNPs (A) and SVs (B). In both matrices, the upper triangle displays the estimated genetic correlations values, while the lower triangle shows their corresponding standard errors. "NA" in panel B indicates pairs where the SV-based model failed to converge. **C & D:** Cluster analysis based on the estimated genetic correlation matrix produced from 15 traits using SNPs (C) and SVs (D).

To investigate the shared genetic architecture among the 15 traits, we estimated pairwise genetic correlations ($r_{g}$) using SNPs (Fig. 3A) and SVs (Fig. 3B) independently. Overall, the correlation patterns revealed by both marker types exhibited a high degree of concordance. BW1 and AFW displayed distinct genetic architectures compared to the other traits. The average SNP-based $r_{g}$ between BW1 and other traits was 0.053, and that for AFW was 0.194. Consistently, the SV-based analysis yielded similar average values of 0.073 for BW1 and 0.198 for AFW. However, a robust cluster of strongly positively correlated traits was identified among the remaining traits, including BW21, BW49, BMW, LMW, HEW, EW, WW, PSLW, SW, and HNW. In the SNP-based analysis, pairwise correlations within this group consistently exceeded 0.75, with most surpassing 0.80. For instance, the $r_{g}$ between EW and WW reached 0.98. This high-correlation structure was well recapitulated by the SV-based analysis (e.g., $r_{g}$= 0.95 for EW and WW). Notably, for certain trait pairs exhibiting near-unity correlation in the SNP analysis (e.g., EW and SW, $r_{g}\approx 1$), the SV-based model failed to converge, resulting in missing values (NA). This likely reflects high multicollinearity or insufficient distinct variance components captured by SVs for these specific highly correlated phenotypes. Furthermore, hierarchical clustering based on estimated genetic correlations (Fig. 3C and 3D) categorized the traits into three major groups: BW1 and AFW formed distinct, separate clusters, while the remaining growth-related traits aggregated into a single major clade. Overall, the genetic correlation analysis suggests that there may be a common underlying genetic basis or physiological mechanism shared among various traits (Lu et al., 2021).

### Integrated SNP and SV GWAS Reveals the Genetic Architecture of Growth and Carcass Traits

Based on single-trait GWAS models result (Fig. S5), we identified three major genome-wide significant SNP peaks that were associated with multiple traits, predominantly located on chromosomes (Chr) 1, 4, and 27. Specifically, the peak on Chr 1 (165.31–175.55 Mb) showed significant associations with all traits except BW1 and AFW. The peak on Chr 4 (71.73–78.98 Mb) was significantly associated with eight traits: LMW, HEW, EW, WW, PSLW, SW, SCW, and HLGGSWs. Similarly, the peak on Chr 27 (2.69–4.26 Mb) was associated with six traits, including LMW, WW, PSLW, SCW, SIL, and HLGGSWs. In addition to these multi-trait loci, a trait-specific significant peak for BW1 was identified on Chr 7 (13.98–13.99 Mb). However, no genome-wide significant signals were detected for AFW. The co-localization of significant signals for multiple traits within the genomic regions on Chr 1, 4, and 27 suggests the presence of QTLs with pleiotropic effects regulating these phenotypes.

**Fig. 4:**
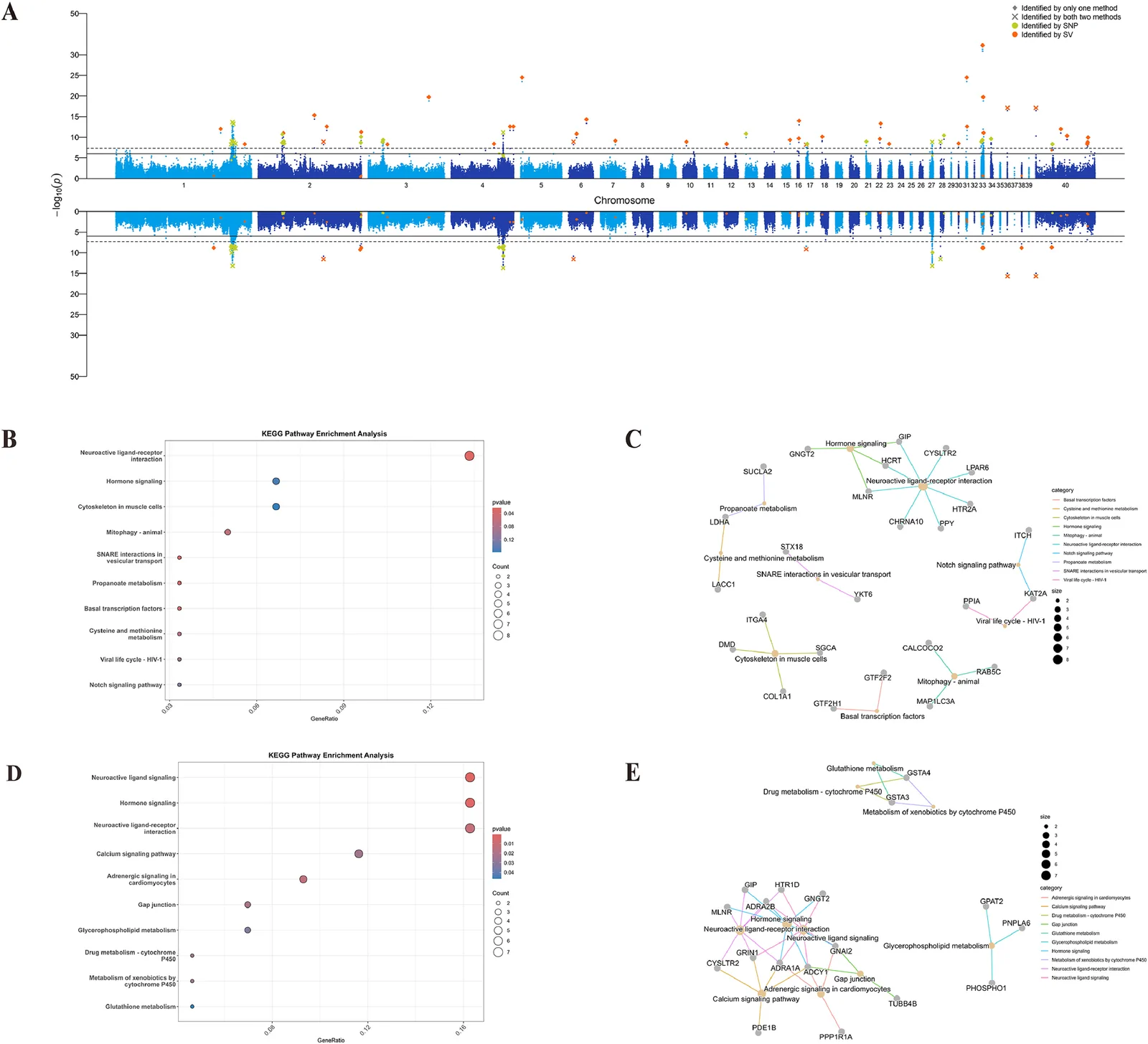
Comparative GWAS and functional enrichment analysis of chicken growth and carcass traits. **A:** Mirror Manhattan plot of C-GWAS and MinGWAS results. The upper panel displays multi-trait association results (C-GWAS), while the lower panel shows the minimum P-values from single-trait GWAS (MinGWAS). The solid horizontal line indicates the suggestive threshold ($1\;\times 10^{-6}$), and the dashed line represents the genome-wide significance threshold ($5\;\times 10^{-8}$). Markers identified by both methods are denoted by crosses, while those unique to one method are represented by diamonds. Red and yellowish-green colors distinguish structural variations (SVs) from SNPs, respectively.. **B–E:** KEGG enrichment analysis for MinGWAS (B, C) and C-GWAS (D, E) candidate genes, visualized via dot plots (B, D) and gene-concept networks (C, E).

Subsequently, we performed a multi-trait GWAS analysis using the C-GWAS framework, integrating summary statistics from the single-trait GWAS. The resulting Manhattan plot is presented in Fig. 4A, where the upper panel displays the p-values from C-GWAS, and the lower panel shows the minimum p-values derived from the single-trait GWAS analyses (MinGWAS). The C-GWAS uncovered a larger set of significant genomic regions compared to MinGWAS. Beyond the major peaks previously identified on chromosomes 1, 4, and 27, C-GWAS revealed additional significant association peaks on chromosomes 2 (Chr 2: 34.67-37.80Mb), 3 (Chr 3: 21.04-21.93Mb), 17 (Chr17: 1.36Mb-2.55Mb), and 33 (Chr33: 1.67Mb-3.60Mb). Furthermore, C-GWAS demonstrated superior sensitivity in detecting structural variants (SVs). At the genome-wide suggestive threshold, C-GWAS identified 713 SNPs and 68 SVs, whereas MinGWAS identified 1,187 SNPs but only 18 SVs. Based on these significant variants, we mapped 149 candidate genes from the C-GWAS results and 168 candidate genes from the MinGWAS results.

Within the SNP peaks identified by both methods, numerous well-known candidate genes were observed. On chromosome 1, we identified *FOXO1, SETDB2, PHF11, ITM2B, SLC25A30, MLNR, NEK3, NEK5, CAB39L, CKAP2, RL11, RCBTB1, KPNA3, TRIM13*, and *RNASEH2B*. The peak on chromosome 4 harbored *NCAPG, LDB2*, and *LCORL*. On chromosome 27, *IGF2BP1, GIP, GNGT2*, and *PHOSPHO1* were identified. Most of these genes have been previously reported to influence growth and carcass traits in chickens (Wang et al., 2020a; Zhang et al., 2021; Wang et al., 2024a; Zhong et al., 2024; Zhu et al., 2025b). Notably, the multi-trait C-GWAS framework detected signals that were missed by the single-trait MinGWAS approach. For instance, within the shared peak on chromosome 27, C-GWAS uniquely identified *CDK12*. Additionally, within the novel SNP peaks revealed exclusively by C-GWAS, we identified *RPL15* and *NR1D2* on chromosome 2; *RPS6KC1, DTL*, and *ATF3* on chromosome 3; and *GRIN1* and *TUBB4B* on chromosome 17.

We identified multiple significant SVs and their corresponding candidate genes that were undetected by MinGWAS. Specific examples include a deletion (DEL) at 5,088,107 bp on chromosome 10 (*ICE2*); a DEL at 3,131,990 bp on chromosome 5 (*FANCF*); a DEL at 36,580,055 bp on chromosome 2 (*ZNF385D*); a duplication (DUP) at 2,453,775 bp on chromosome 18 (*MYH10*); a DEL at 11,511,115 bp on chromosome 6 (*STOX1*); a DEL at 49,635,389 bp on chromosome 4 (*MOB1B*); a DEL at 27,966,198 bp on chromosome 3 (*LRFN2*); and an inversion (INV) at 4,405,306 bp on chromosome 22 (*ADRA1A*).

To elucidate the biological functions of the candidate genes identified by the two methods, we performed KEGG pathway enrichment analysis. As shown in Fig. 4B-C, the genes identified by MinGWAS were primarily enriched in pathways such as *Neuroactive ligand-receptor interaction, Hormone signaling*, and *Cytoskeleton in muscle cells*. Conversely, the candidate genes detected by C-GWAS showed enrichment in a broader and more diverse set of signaling and metabolic pathways (Fig. 4D-E), including *Neuroactive ligand signaling, Hormone signaling, Neuroactive ligand-receptor interaction, Calcium signaling pathway, Adrenergic signaling in cardiomyocytes, Gap junction,* and *Glycerophospholipid metabolism*.

### Integrated gene-level analysis and eQTL colocalization identify key pleiotropic regulators of growth and carcass traits

**Fig. 5:**
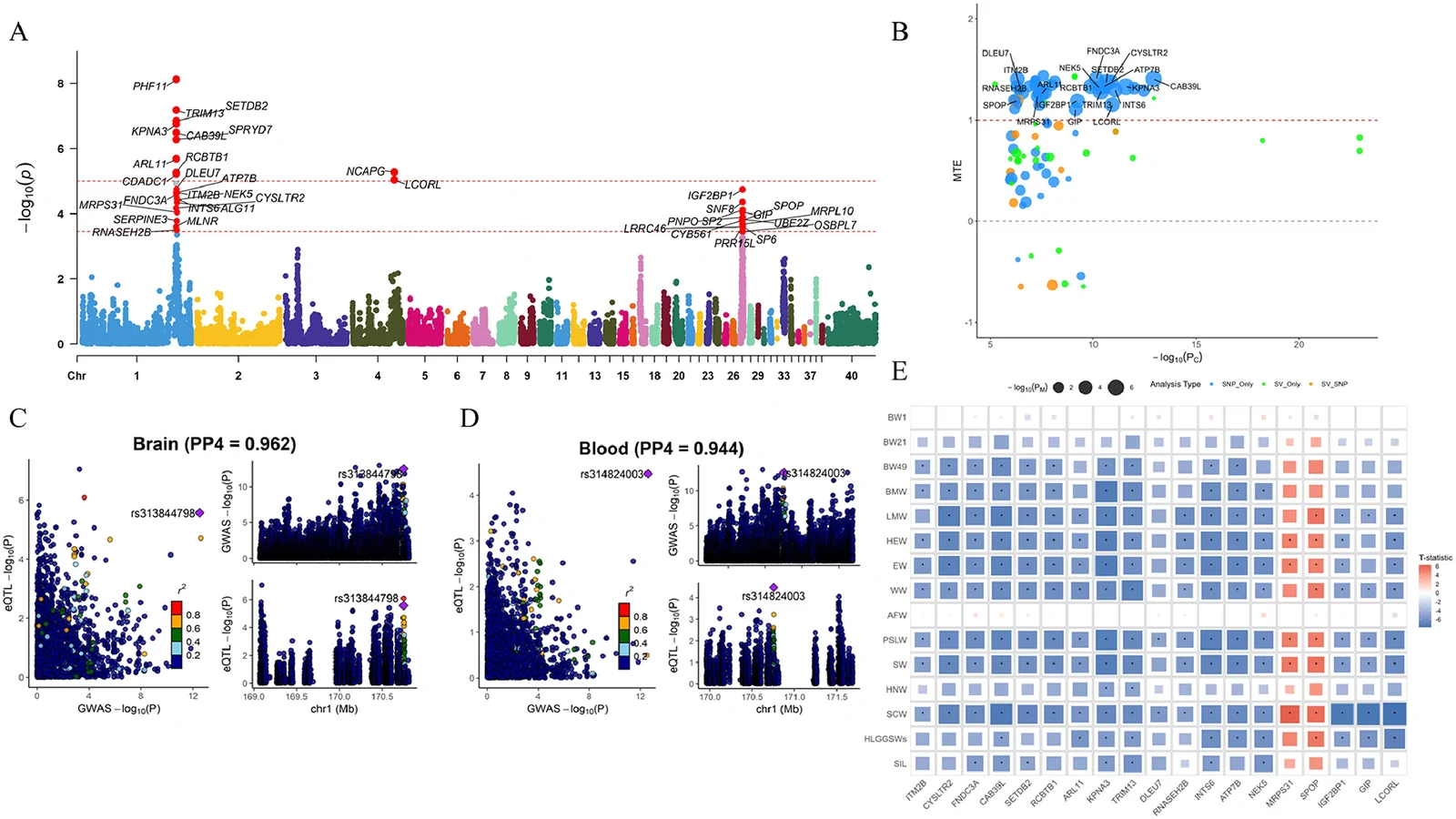
Identification and functional validation of key pleiotropic regulators for chicken growth and carcass traits.**A:** Gene-based association analysis. Manhattan-style plot showing the significance of genes derived from the multi-trait C-GWAS framework. The red dashed lines represent the significance thresholds ($P<1\;\times 10^{-5}$ and $P<3.5\;\times 10^{-4}$). **B:** Prioritization of candidate genes using multi-trait effect (MTE). Scatter plot illustrates the relationship between C-GWAS significance ($-log{}_{10}P_{c}$, x-axis) and MTE values (y-axis). Representative genes (MTE > 1, C-GWAS $P<1\;\times 10^{-6}$, and gene-based $P<3.5\;\times 10^{-4}$) are labeled, with bubble size indicating the magnitude of significance for gene-based association analysis. **C & D**: Colocalization of C-GWAS and tissue-specific eQTL signals for *ITM2B* in brain (C) and *TRIM13* in blood (D). The color scale indicates the linkage disequilibrium ($r^{2}$) with the lead variant (purple diamond). **E:** Pleiotropic profile of 19 representative genes across 15 traits. Dot size and color represent effect (T-statistic), while black dots denote associations surpassing threshold ($1\;\times 10^{-6}$).

To further explore genetic significance at the gene level, we performed a gene-based association analysis using the summary statistics derived from the multi-trait C-GWAS framework (Fig. 5A). At a significance threshold of $p<1\;\times 10^{-5}$, 11 genes were identified on chromosomes 1 and 4. Specifically, the significant genes on chromosome 1 included *PHF11, TRIM13, SETDB2, CDADC1, CAB39L, RCBTB1, ARL11, KPNA3*, and *SPRYD7*, while *LCORL* and *NCAPG* were identified on chromosome 4. All these genes have been previously reported to be associated with growth or carcass traits in chickens (Wang et al., 2020b; Liu et al., 2021; Li et al., 2022a; Wang et al., 2024a; Jiang et al., 2025). When the threshold was expanded to $p<3.5\;\times 10^{-4}$, a total of 36 candidate genes were detected. Aside the highlighted genes above, *ITM2B, MLNR*, and *RNASEH2B* were identified on chromosome 1, and *SPOP, IGF2BP1*, and *GIP* were found on chromosome 27. *IGF2BP1* emerged remarkably as the most significant gene on chromosome 27, consistent with its well-documented role in regulating growth and carcass traits(Zhou et al., 2018; Wang et al., 2021; Zhu et al., 2025a).

Subsequently, we employed the method of study to calculate the multi-trait effect (MTE) of the genes annotated within the C-GWAS framework. The MTE was calculated based on the most significant loci mapped to each gene. In cases where the most significant locus was annotated to more than two genes simultaneously, the gene closest to that locus was selected as the representative gene for that locus. For example, on chromosome 4, the lead SNP for *LCORL* and *NCAPG* was identical; consequently, *LCORL* was selected as the representative gene for that locus. Fig. 5B highlighted genes that prioritized specific stringent criteria, including a C-GWAS $p<1\;\times 10^{-6}$, a gene-based association $p<3.5\;\times 10^{-4}$, and an MTE > 1. Out of the 29 genes surpassing these thresholds, 19 representative genes were labeled in figure.

We performed a colocalization analysis for these 29 genes by integrating the C-GWAS summary data with tissue-specific eQTLs obtained from the Chicken GTEx database. We identified three significant colocalization events with a posterior probability of colocalization (PP4) > 0.85. Specifically, the C-GWAS QTL signal significantly colocalized with the eQTL of *ITM2B* in brain tissue (PP4=0.962), with the lead colocalization signal pointing to rs313844798 (Fig. 5C). *ITM2B* encodes a widely expressed type II transmembrane protein often associated with neurodegeneration in humans (Martins et al., 2021), and mouse models have further confirmed its physiological role in regulating excitatory synaptic transmission (Yao et al., 2019). A significant colocalization was also observed between the C-GWAS signal and the eQTL of *TRIM13* in blood (PP4=0.944), with rs314824003 as the lead colocalization signal (Fig. 5D). *TRIM13* encodes an endoplasmic-reticulum-associated E3 ubiquitin ligase (Li et al., 2022b), but its potential role in regulating growth traits in chicken requires further functional validation. In liver tissue, the C-GWAS signal colocalized with the eQTL of *CAB39L* (PP4=0.875), again pointing to rs313844798 (Fig. S6). *CAB39L* encodes a calcium-binding protein involved in intracellular calcium signaling and has been reported to mediate phosphorylation processes that activate the AMPK signaling pathway which regulates feeding behavior (Minokoshi et al., 2004; Zhang et al., 2021). The variant rs313844798 colocalized with both *ITM2B* in brain tissue and *CAB39L* in liver tissue. This "pleiotropic" colocalization signal suggests that this locus may drive complex phenotypes by regulating different target genes across various tissues.

To further validate the genetic effects of these candidate genes, we evaluated the single-trait GWAS significance and T-statistics for the 19 representative genes across all analyzed traits (Fig. 5E). All 19 genes were significantly associated with at least two traits. Remarkably, approximately 79% (15/19) of these genes showed significant associations with eight or more traits. The three colocalized genes namely *TRIM13, CAB39L*, and *ITM2B* were closely associated with 12, 11, and 8 growth or carcass traits, respectively. This broad pattern of cross-trait association strongly indicates that these candidate genes possess pleiotropic effects and play a central role in regulating growth and development.

### Enhanced genomic prediction accuracy through synergistic integration of SNPs and SVs

**Fig. 6:**
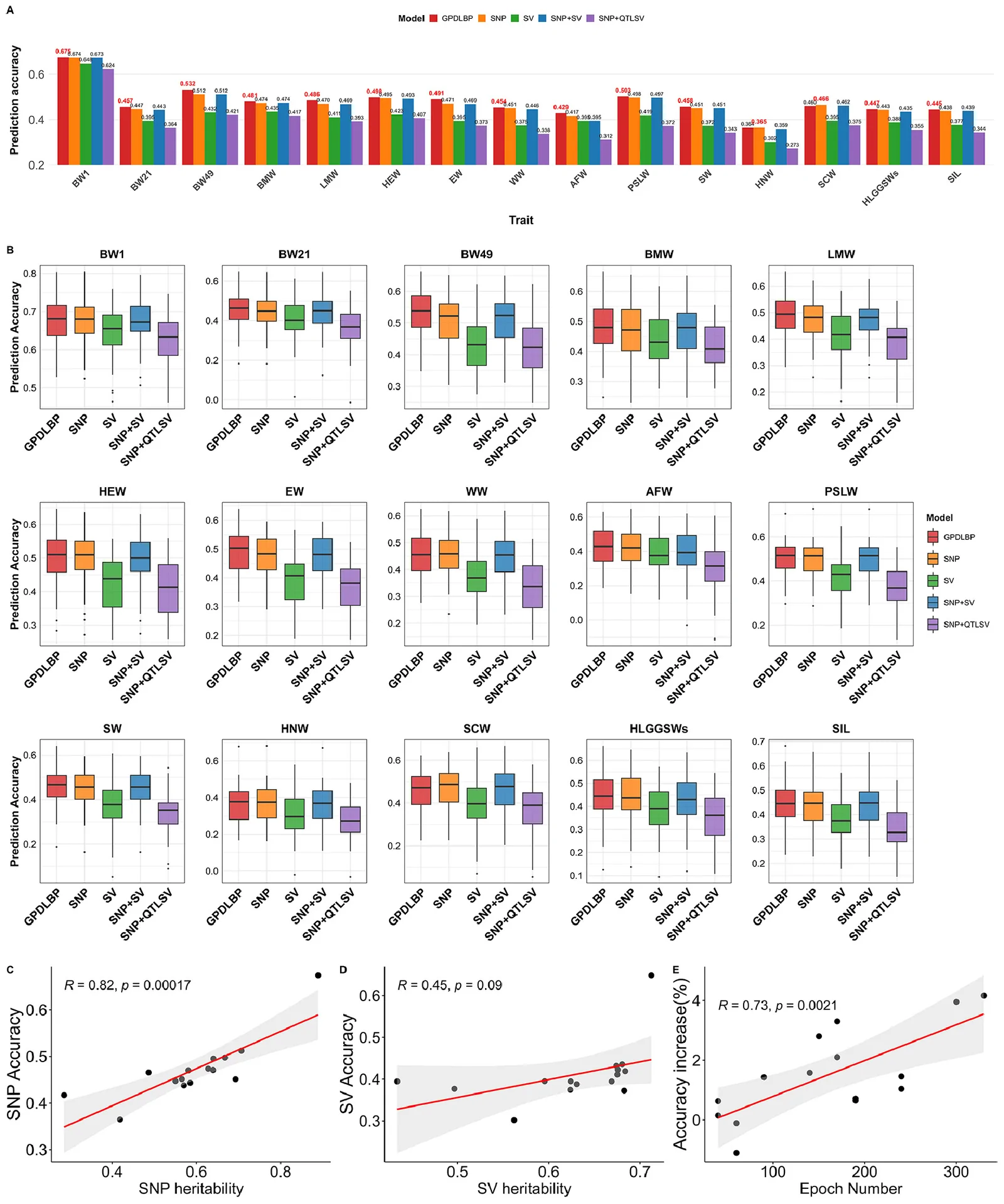
**Genomic prediction performance of different models across 15 traits. A:** Comparison of average genomic prediction accuracy among five models. Numerical values for accuracy are labeled atop the bars. **B.** Box plots show the distribution of prediction accuracies across cross-validation folds for each trait and model. **C & D.** Correlation analysis between estimated genomic heritability ($h^{2}$) and prediction accuracy for SNPs (C) and SVs (D). **E.** Correlation analysis between the accuracy gain (%) achieved by GPDLBP (relative to the SNP-only model) and the number of training epochs required for model convergence.

Building on the assessment of SNPs and SVs using traditional linear models, we developed GPDLBP, a unified framework designed to effectively integrate SNPs and SVs for enhanced genomic prediction. The comparative performance of genomic prediction is illustrated in Fig. 6, with the average prediction accuracy shown in Fig. 6A and the distribution of accuracy across cross-validation presented in Fig. 6B. The mean and standard deviation of prediction accuracy across cross-validation are reported in Table S11. Among the analyzed traits, AFW exhibited the lowest genomic prediction accuracy, while BW1 showed the highest. In the baseline comparison using linear models, prediction accuracy using SVs alone was consistently lower than that using SNPs alone across all traits. Notably, the straightforward integration of both markers within multi-component linear model (SNP+SV) did not result in the expected performance improvement compared to the SNP-only model; in some cases, the accuracy even slightly decreased. Furthermore, simply incorporating the prior QTL-associated SVs (QTLSV) identified by GPDLBP back into a multi-component linear model (SNP+QTLSV) resulted in the poorest performance, underscoring the limitations of linear models in capturing these complex effects. Contrarily, the GPDLBP framework achieved consistent improvements in prediction accuracy for the most traits. Modest but statistically significant improvements were observed particularly for BW21, BW49, EW, LMW, and AFW, where the average prediction accuracy increased by over 2% compared to the SNP-only model (FDR < 0.05; Fig. S7). For instance, GPDLBP increased the average accuracy from 0.512 to 0.532 for BW49, and from 0.471 to 0.491 for EW. To investigate the factors influencing prediction accuracy, we analyzed the relationship between trait heritability and model performance. A significant positive correlation was observed between SNP-based heritability and genomic prediction accuracy (R=0.82,p=0.00017, Fig. 6C), consistent with the expectation that traits with higher heritability generally yield higher genomic prediction accuracy. However, the correlation between SV-based heritability and accuracy was moderate and not statistically significant (R=0.45,p=0.09, Fig. 6D). Intriguingly, we identified a significant positive correlation between the accuracy gain achieved by GPDLBP (relative to the SNP-only model) and the optimal number of training epochs (R=0.73,p=0.0021, Fig. 6E).

## Discussion

Although recent genomic studies in chickens have highlighted the substantial impact of genomic variants on quantitative traits (Yang et al., 2021b; Zhong et al., 2024), these efforts have predominantly focused on SNPs, leaving structural variants SVs largely unexplored. In the present study, we integrated genome-wide SNP and SV data to provide a comprehensive assessment of the genetic architecture underlying key economic traits in chickens. Furthermore, we introduced GPDLBP, a novel deep learning framework that synergistically combines SNPs and SVs information to enhance genomic prediction accuracy.

Among current short-read sequencing algorithms, four primary strategies are employed for SV calling: split-read mapping, discordant read-pair analysis, read-depth variation, and de novo genome assembly. Each strategy exhibits distinct strengths and limitations, contingent upon the variant type and the sequence architecture underlying the SV locus (Cameron et al., 2019; Kosugi et al., 2019). By requiring concordant support from at least two independent callers, our consensus framework was designed to reduce caller-specific false-positive SVs and generate a relatively conservative SV dataset. However, this increase in specificity may be accompanied by reduced sensitivity because true variants detected by only one caller may be excluded. This limitation may be particularly relevant for rare SVs, variants in repetitive or complex genomic regions, and SV types for which the four callers have different detection sensitivities. Moreover, the consensus strategy may preferentially retain SVs that are readily detected by multiple algorithms. Therefore, the resulting dataset should be interpreted as a conservative consensus SV set rather than a comprehensive catalog of all structural variation in this population. Previous evidence suggests that integrating outputs from multiple tools—rather than strictly restricting analysis to overlap—can maximize performance and enhance sensitivity to discover novel SV loci (Zong et al., 2023). Our findings indicate that deletions (DELs) are the predominant SV type in the chicken genome, followed by duplications (DUPs), which is consistent with previous reports. Similar patterns have been observed in other livestock species, such as cattle and pigs (Chen et al., 2021; Zong et al., 2023), where DELs constitute the majority of the SV catalog and account for the vast majority of genes identified through SV-GWAS. These observations suggest that DELs are not only the most widely distributed SV type in livestock genomes but also exert more profound genetic effects on complex traits and exhibit superior detection reliability compared to other SV categories.

Compared to SNPs, structural variations (SVs) are more likely to modulate gene expression and phenotypic variation through dosage effects or by altering gene copy number (Fudenberg and Pollard, 2019; Jakubosky et al., 2020; Alonge et al., 2020). In the present study, the relative contribution of SNPs and SVs to heritability estimates varied according to the trait-specific genetic architecture. However, in contrast to using single marker types in isolation, the integration of both SNPs and SVs yielded superior heritability estimates for most traits (with the exception of AFW). This observation aligns with previous findings in humans (GTEx Consortium et al., 2017), tomatoes (Alonge et al., 2020), and sorghum (Zhang et al., 2024). These studies suggest that the combined analysis of SVs and SNPs enhances the identification of putative expression quantitative trait loci (eQTLs). Furthermore, the direct impact of SVs on gene expression likely represents a critical mechanism for resolving the "missing heritability" in complex trait analyses (Zhang et al., 2024).

In this study, the single-trait GWAS results revealed significant SNP peaks on chromosomes 1, 4, and 27. The association region on chromosome 1 (165.31–175.55 Mb) encompasses a dense cluster of candidate genes associated with growth and carcass traits in chicken, such as *FOXO1, SETDB2, PHF11, MLNR, CAB39L, ITM2B*, and *TRIM13*. This specific genomic interval on Chr1 is well-documented as a major QTL for growth traits across diverse chicken populations (Wang et al., 2020b; Zhu et al., 2025b). On chromosome 4 (71.73–78.98 Mb), the significant signal encompassed the *NCAPG-LCORL* locus, a widely recognized "master regulator" of body size across multiple species (Takasuga, 2016; Wyler et al., 2024; Bai et al., 2025). Furthermore, the association peak on chromosome 27 contains *IGF2BP1*, which has recently emerged as a primary candidate gene influencing growth in chickens (Wang et al., 2021, 2024a). Indeed, Zhong et al. (Zhong et al., 2024) demonstrated that the eQTL signals for *IGF2BP1* are significantly associated with body weight at specific developmental stages in poultry. Compared to conventional single-trait GWAS (MinGWAS), the implementation of a multi-trait C-GWAS framework significantly enhances the statistical power to detect association signals (Xiong et al., 2022). C-GWAS uncovered additional association peaks on chromosomes 2, 3, 17, and 33. On chromosome 2, the candidate gene *RPL15*, a member of the ribosomal protein (RP) family, serves as a fundamental component of the protein synthesis machinery. Wen et al. (Wen et al., 2016) suggested that ribosomal biogenesis is a prerequisite and rate-limiting step for skeletal muscle hypertrophy, as translational capacity directly dictates the accumulation of muscle mass. On chromosome 3, *ATF3* emerged as a key regulator of glucose homeostasis and metabolism (Hu et al., 2023). Previous findings have shown its differential expression in chicken liver and muscle tissues under varying dietary protein levels (Kurniawan et al., 2023). On chromosome 17, *GRIN1* encodes the essential GluN1 subunit of the NMDA receptor, which is highly expressed in the hypothalamus. Research in chickens indicates that NMDA receptor-mediated central glutamatergic signaling plays a pivotal role in orchestrating appetite and feeding behavior (Taati et al., 2011). Additionally, on chromosome 27, C-GWAS identified key candidate, *CDK12*, which has been implicated in osteoblast differentiation and body size variation in ponies (Han et al., 2025).

Moreover, C-GWAS demonstrated superior sensitivity in identifying complex structural variants (SVs). Among the significant SVs detected exclusively by C-GWAS, several genes with potential impacts on growth and carcass traits were identified. For instance, ZNF385D (identified via a DEL on Chr 2) has been linked to body weight and size traits in goats and pigs (Zhang et al., 2022b; Zhou et al., 2025), as well as adaptive phenotypic differentiation in chickens (Tolone et al., 2023). MYH10 (identified via a DUP on Chr 18), encodes a non-muscle myosin involved in actin-dependent motor processes (Conti and Adelstein, 2008). Studies in pigs (Ayuso et al., 2016), cattle (Zhuang et al., 2020), and chickens (Xue et al., 2017) have highlighted *MYH10* as a critical candidate for muscle development across various growth stages. On chromosome 4, *MOB1B* (identified via a DEL) is a core component of the Hippo signaling pathway. Functional studies in mouse models have characterized *MOB1B* as a negative regulator of skeletal muscle differentiation (Zhang et al., 2022a), where its downregulation is essential for the efficient differentiation of C2C12 cells. Moreover, *MOB1B* has been reported as a candidate for growth traits in sheep (Zhang et al., 2025), evidence in other farm animals remains scarce. Finally, compared to MinGWAS, the KEGG enrichment results of C-GWAS exhibited a stronger emphasis on signal transduction pathways. This may suggest that the shared genetic architecture of growth and carcass traits in chickens is governed by signaling pathways that coordinate development across diverse tissues.

To gain deeper insights into the genetic pleiotropy underlying complex traits at the gene level, we conducted a gene-based association analysis using summary statistics derived from the multi-trait C-GWAS framework. At the gene level, our results robustly identified several “star” genes situated at the core of avian growth and carcass trait regulation. Specifically, we identified a potent genomic cluster on chromosome 1 comprising *SETDB2, ARL11, MLNR, CAB39L, ITM2B*, and *TRIM13*, alongside the canonical growth-related loci *NCAPG/LCORL* and *IGF2BP1* on chromosomes 4 and 27, respectively. Integrating Multi-Trait Effect (MTE) analysis with single-trait T-statistics revealed that these genes exert shared genetic effects across traits with diverse architectures (Xiong et al., 2022). This pervasive pleiotropic pattern identifies these loci as 'genetic hubs' that drive complex phenotypic variation by orchestrating various metabolic and developmental pathways. These genes may emerge as 'core nodes' within the regulatory network of chicken development, representing high-priority targets for the synchronized improvement of multiple economic traits in breeding programs.

Furthermore, we performed colocalization analysis by integrating C-GWAS summary statistics with tissue-specific eQTL data from the Chicken GTEx resource. The C-GWAS signal at the locus containing rs313844798 showed strong colocalization evidence with the brain eQTL signal for *ITM2B* and the liver eQTL signal for *CAB39L*. These findings prioritize *ITM2B* and *CAB39L* as candidate genes and suggest that the growth-associated locus may contain tissue-specific regulatory signals. *CAB39L* encodes a scaffold protein involved in AMPK signaling (Minokoshi et al., 2004; Zhang et al., 2021), whereas *ITM2B* has been implicated in the regulation of excitatory synaptic transmission in studies of humans and mice (Yao et al., 2019; Martins et al., 2021). In chickens, *ITM2B* was previously reported to be differentially expressed between groups with divergent body weights (Wang et al., 2020b), providing additional support for its consideration as a candidate growth-related gene. However, these functional annotations and colocalization results do not establish the mechanisms through which either gene may influence chicken growth, nor do they demonstrate coordinated regulation between the brain and liver. Further tissue-specific functional studies are required to determine the biological relevance of these candidate regulatory associations.

In genomic prediction based on conventional linear models, we observed that SNP-only models achieved superior performance. Notably, incorporating both SNPs and SVs into a multi-component linear mixed model failed to enhance prediction accuracy as expected, and in some cases, led to a marginal decrease. This observation aligns with previous studies suggesting that the simply simultaneous inclusion of diverse variant types within traditional linear framework does not necessarily yield additional predictive gains (Chen et al., 2021; Zhuang et al., 2023). Furthermore, while SNP-based prediction accuracy showed a significant positive correlation with estimated heritability consistent with genetic expectations (Xiang et al., 2019), no such correlation was observed for SV-based models in our study. This discrepancy likely reflects the genomic differences between the two markers: unlike the vast and evenly distributed SNPs, SVs frequently exhibit non-uniform genomic distribution and potentially harbor complex non-additive genetic effects, thereby deviating from the additive assumptions underlying conventional linear models.

To address this challenge, we developed GPDLBP, a unified framework that synergistically combines deep learning (DL) networks with the GBLUP model. In this hybrid framework, the GBLUP module accounts for the additive SNP effects, while the DL component, utilizing Locally Connected Neural Networks (LCNNs), is specifically designed to capture the complex genetic effects of SVs. Unlike typical Convolutional Neural Networks (CNNs) that rely on weight-sharing to identify patterns across sliding window (Zingaretti et al., 2020), LCNNs employ non-shared weights across filters (Pook et al., 2020). This allows the model to learn independent weight sets for neurons at different genomic positions, enabling the extraction of position-specific local features that reflect the unique functional heterogeneity of each genomic region (Lee et al., 2023). A key innovation of GPDLBP is feature grouping strategy. Unlike traditional LCNNs that partition fixed physical regions (Lee et al., 2023), GPDLBP first prioritizes "QTL_SVs" based on their genetic contributions and groups them according to their effect intensities. This strategy integrates prior associated SVs into the neural network, assigning variants with similar genetic contributions to the same neuronal group for non-linear feature extraction. This strategy enhances the model's ability to capture the potential nonlinear interactions. This functional partitioning strategy reference to the work of Wang et al. (Wang et al., 2024b), who utilized a partially connected feedforward neural network based on gene-annotated SNP sets to achieve superior predictive performance in cattle compared to traditional GBLUP. Notably, the SNP+QTLSV model performed worse than the SNP-only model across most traits. This finding suggests that within a rigid linear framework, simply adding significant SV loci may introduce bias due to parameter redundancy or model misspecification rather than providing predictive gains. Conversely, GPDLBP processes SV information asynchronously via LCNNs and performs feature fusion at higher layers, offering a more flexible integration of these critical variants. Furthermore, our results revealed a significant positive correlation between accuracy gains and the optimal number of training epochs, suggesting that deeper iterative optimization is essential for unearthing latent non-linear signals within complex genomic architectures. We postulated that the increase in epoch requirements reflects the iterative depth necessary for the model to excavate deep non-linear features. For traits exhibiting substantial improvements, such as BW49 and EW, the model requires extended optimization cycles to capture latent genetic signals embedded within complex genomic architectures. This suggests that GPDLBP, through prior grouping and localized weighting structure, may capture additional predictive signals beyond the linear SNP-only model, provided that feature selection and model training are performed within a leakage-free validation framework. In conclusion, by incorporating genetic prior grouping strategy and non-shared weight LCNNs architectures, the GPDLBP framework outperforming traditional linear models in genomic prediction across most traits. We anticipate that this methodology will provide a robust theoretical framework for the efficient utilization of structural variants in genome prediction, particularly in the context of high-depth whole-genome resequencing, ultimately accelerating precision breeding.

We acknowledge that GPDLBP has several limitations. First, SNPs and SVs may be in linkage disequilibrium, and the corresponding SNP-GRMs and SV-based GRMs may therefore capture partially overlapping genetic signals. Because the covariance between these two components was not explicitly modeled in the present two-component framework, the estimated variance attributed to SVs and their predictive contribution should be interpreted with caution. Second, the relatively modest sample size may increase the risk of overfitting in the deep-learning component. Consequently, the observed performance of GPDLBP should be regarded as preliminary until it is confirmed in larger populations with a more favorable ratio of sample size to model complexity. Third, the predictive performance of GPDLBP was evaluated using repeated random cross-validation within a single F2 population, without validation in an independent population. Therefore, the present results demonstrate the within-population predictive utility of GPDLBP, rather than its generalizability to other chicken populations or breeding programs. Although GPDLBP provided statistically significant improvements for several traits, the absolute increases in prediction accuracy were modest. Higher prediction accuracy may theoretically enhance selection response, but such a modest improvement does not necessarily imply a practically meaningful increase in realized genetic gain. Its breeding value will depend on the trait, population, selection scheme, available genetic variation, and implementation costs. Therefore, independent population validation and prospective selection experiments are required to determine whether the observed improvements translate into measurable genetic gain.

## Conclusion

Using high-depth whole-genome resequencing data (average coverage > 30 ×), this study systematically identified SNPs and SVs and characterized the genetic architecture of growth and carcass traits in chickens at high resolution. To better incorporate structural variation in genomic selection, we developed GPDLBP, a deep learning framework that integrates SNP and SV information. Our results demonstrate that the GPDLBP framework improves genomic prediction accuracy across most traits in chicken. Overall, this study highlights the potential value of integrating multiple classes of genomic variants for high-depth sequence-informed genomic evaluation and poultry breeding.

## Authors’ Contributions

QHN and WL conceived the study and designed the experiments. HQY and SYZ performed statistical analysis, visualized the data, and wrote the manuscript. LQ performed data processing. XQL, SFB, CBZ performed edited and revised the manuscript. QHN revised the final manuscript. All authors reviewed and agreed to the published version of the manuscript.

## Ethics Approval

This study was conducted according to the guidelines of the Declaration of Helsinki and approved by South China Agricultural University Institutional Animal Care and Use Committee (Approval number: SCAU#2022A031). The project was approved on August 29, 2022.

## Funding Information

This work was supported by the Biological Breeding-National Science and Technology Major Project (2023ZD04064), National Key R&D Program of China (2021YFD1300100) and China Agriculture Research System of MOF and MARA (CARS-41), the Science and Technology Program of Guangdong province (2023B1212060057), the the Project of the Seed Industry Revitalization of Department of Agriculture (2024-XPY-00-003), and Innovation Youth Science Fund Project of Fujian Provincial Natural Science Foundation (2026J008276).

## Availability of Data and Materials

The source code for the GPDLBP algorithm is publicly available on GitHub at https://github.com/ HaoqiangYe/GPDLBP. The sequenced datasets used in this study are available from the corresponding authors upon reasonable request.

## Disclosures

The authors have declared that no competing interests exist.

## Appendix Supplementary materials

Supplementary Data Supplementary Fig. 1. Summary of identified SV counts per type. Supplementary Fig. 2. Permutation-based enrichment of genes and structural variants across genomic regions. Fold enrichment of genes and structural variants (SVs) was evaluated in chromosome-end, gene-rich, and gene-poor regions. Chromosome-end regions were defined as the terminal 5% at each end of each chromosome, whereas gene-rich and gene-poor regions were defined according to the upper and lower quartiles of gene density in non-overlapping 1-Mb genomic windows, respectively. Points represent fold enrichment, calculated as the ratio of the observed overlap count to the mean overlap count obtained from 10,000 within-chromosome permutations. Horizontal lines connect the null expectation of 1 to the observed fold-enrichment estimate, and the dashed vertical line indicates random expectation (fold enrichment = 1). Red points indicate significant enrichment after Benjamini–Hochberg correction, whereas gray points indicate nonsignificant results. Numerical labels show fold-enrichment values. INS, insertion; INV, inversion; DUP, duplication; DEL, deletion; All_SVs, all structural variants combined. *FDR < 0.05; **FDR < 0.01; ***FDR < 0.001. Supplementary Fig. 3. A & B: Principal component analysis based on the SNP (A) and SV (B). C & D: Heatmaps of genomic kinship matrices estimated using SNPs (C) and SVs (D). E: Visualization of the density of the number of SV $r^{2}$ ranks surpassing the SNP-based median $r^{2}$ values. F: Comparison of MAF distributions between relatively low-LD and high-LD SV groups. Significant differences were tested by two-tailed Wilcoxon test (**p<0.01). Supplementary Fig. 4. Distributions of linkage disequilibrium ($R^{2}$) between structural variants and nearby SNPs. A: Distribution of the maximum SV–SNP $R^{2}$ value for each SVs. B: Distribution of the mean SV–SNP $R^{2}$ value for each SVs. Supplementary Fig. 5. Circular Manhattan plots of single-trait GWAS results for 15 growth and carcass traits. Each concentric track represents the genome-wide association results for a specific trait. Traits are arranged concentrically from the innermost to the outermost ring in the following order: BW1, BW21, BW49, BMW, LMW, HEW, EW, WW, AFW, PSLW, SW, HNW, SCW, HLGGSWs, and SIL. BW1, body weight at birth; BW21, body weight at 21 days of age; BW49, body weight at 49 days of age; BMW, breast muscle weight; LMW, leg muscle weight; HEW, half eviscerated weight; EW, eviscerated weight; WW, wing weight; AFW, abdominal fat weight; PSLW, pre-slaughter live weight; SW, slaughter weight; HNW, head and neck weight; SCW, shank and claw weight; HLGGSWs, heart-liver-gizzard-glandular-stomach weight; SIL, small intestine length. Supplementary Fig. 6. Colocalization of C-GWAS and tissue-specific eQTL signals for *CAB39L* in liver. Supplementary Fig. 7. Differences in prediction accuracy between GPDLBP and SNP-only GBLUP. Points indicate the mean paired difference in prediction accuracy across matched cross-validation replicates, calculated as GPDLBP minus SNP-only GBLUP, and horizontal lines represent the corresponding 95% confidence intervals. The dashed vertical line indicates no difference between the two models. Statistical significance was assessed using paired t-tests, with P values adjusted across traits using the Benjamini–Hochberg procedure. *, FDR < 0.05; ***, FDR <0.001; ns, not significant. Image, application 1

Supplementary Table 1. The descriptive statistics for 15 growth and carcass traits in chicken population. Supplementary Table 2. Structural variant detection by caller. Supplementary Table 3. The Genome-wide significant SNPs identified by C-GWAS. Supplementary Table 4. The Genome-wide significant SVs identified by C-GWAS. Supplementary Table 5. The Genome-wide significant SNPs identified by MinGWAS. Supplementary Table 6. The Genome-wide significant SVs identified by MinGWAS. Supplementary Table 7. The candidate genes were annotated by significant variants identified by C-GWAS. Supplementary Table 8. The candidate genes were annotated by significant variants identified by MinGWAS. Supplementary Table 9. KEGG pathway enrichment analysis for candidate genes from C-GWAS. Supplementary Table 10. KEGG pathway enrichment analysis for candidate genes from MinGWAS. Supplementary Table 11. The mean and standard deviation of prediction accuracy across cross-validation Image, application 2

## Footnotes

Supplementary material associated with this article can be found, in the online version, at doi:10.1016/j.psj.2026.107561.
