# Supplementary material for: Integrated Analysis of SNPs and Structural Variations via High-depth Whole-genome Sequencing Reveals the Genetic Architecture and Optimizes Genomic Prediction in Chickens
Source: Poult Sci. 2026 Aug 3;105(11):107561. doi: 10.1016/j.psj.2026.107561 (PMC13499392; doi:10.1016/j.psj.2026.107561)
Supplement: Supplementary file 1 [file mmc1.docx]

**Supplementary Figure 1.** Summary of identified SV counts per type.

**Supplementary Figure 2. Permutation-based enrichment of genes and structural variants across genomic regions.** Fold enrichment of genes and structural variants (SVs) was evaluated in chromosome-end, gene-rich, and gene-poor regions. Chromosome-end regions were defined as the terminal 5% at each end of each chromosome, whereas gene-rich and gene-poor regions were defined according to the upper and lower quartiles of gene density in non-overlapping 1-Mb genomic windows, respectively. Points represent fold enrichment, calculated as the ratio of the observed overlap count to the mean overlap count obtained from 10,000 within-chromosome permutations. Horizontal lines connect the null expectation of 1 to the observed fold-enrichment estimate, and the dashed vertical line indicates random expectation (fold enrichment = 1). Red points indicate significant enrichment after Benjamini–Hochberg correction, whereas gray points indicate nonsignificant results. Numerical labels show fold-enrichment values. INS, insertion; INV, inversion; DUP, duplication; DEL, deletion; All_SVs, all structural variants combined. $*$FDR $<$ 0.05; $**$FDR $<$ 0.01; $***$FDR $<$ 0.001.

**Supplementary Figure 3.** **A & B**: Principal component analysis based on the SNP (A) and SV (B). **C & D**: Heatmaps of genomic kinship matrices estimated using SNPs (C) and SVs (D). **E:** Visualization of the density of the number of SV $r^{2}$ ranks surpassing the SNP-based median $r^{2}$ values. **F:** Comparison of MAF distributions between relatively low-LD and high-LD SV groups. Significant differences were tested by two-tailed Wilcoxon test ($**p<0.01$).

**Supplementary Figure 4. Distributions of linkage disequilibrium (**$\boldsymbol{R}^{\boldsymbol{2}}$**) between structural variants and nearby SNPs.** A: Distribution of the maximum SV–SNP $R^{2}$ value for each SVs. B: Distribution of the mean SV–SNP $R^{2}$ value for each SVs.

**Supplementary Figure 5.** Circular Manhattan plots of single-trait GWAS results for 15 growth and carcass traits. Each concentric track represents the genome-wide association results for a specific trait. Traits are arranged concentrically from the innermost to the outermost ring in the following order: BW1, BW21, BW49, BMW, LMW, HEW, EW, WW, AFW, PSLW, SW, HNW, SCW, HLGGSWs, and SIL. BW1, body weight at birth; BW21, body weight at 21 days of age; BW49, body weight at 49 days of age; BMW, breast muscle weight; LMW, leg muscle weight; HEW, half eviscerated weight; EW, eviscerated weight; WW, wing weight; AFW, abdominal fat weight; PSLW, pre-slaughter live weight; SW, slaughter weight; HNW, head and neck weight; SCW, shank and claw weight; HLGGSWs, heart-liver-gizzard-glandular-stomach weight; SIL, small intestine length.

**Supplementary Figure 6.** Colocalization of C-GWAS and tissue-specific eQTL signals for *CAB39L* in liver.

**Supplementary Figure 7.** Differences in prediction accuracy between GPDLBP and SNP-only GBLUP. Points indicate the mean paired difference in prediction accuracy across matched cross-validation replicates, calculated as GPDLBP minus SNP-only GBLUP, and horizontal lines represent the corresponding 95% confidence intervals. The dashed vertical line indicates no difference between the two models. Statistical significance was assessed using paired t-tests, with $P$ values adjusted across traits using the Benjamini–Hochberg procedure. *, FDR $<$ 0.05; ***, FDR $<$0.001; ns, not significant.
